# Can small bowel obstruction during pregnancy be treated with conservative management? A review

**DOI:** 10.1186/s13017-024-00541-y

**Published:** 2024-04-10

**Authors:** Xiao Shuang Ling, Wei Cheng Anthony Brian Tian, Goran Augustin, Fausto Catena

**Affiliations:** 1 Department of General Surgery, Changi General Hospital, Singapore, Singapore; 2 Department of General Surgery, Singapore General Hospital, Singapore, Singapore; 3 Department of Surgery, University Hospital Centre Zagreb, Zagreb, Croatia; 4 General and Emergency Surgery Department, Bufalini Hospital, Cesena, Italy

**Keywords:** Small bowel obstruction, Pregnancy, Total parenteral nutrition

## Abstract

**Background:**

Small bowel obstruction can occur during pregnancy, which, if missed, can lead to dire consequences for both the mother and foetus. Management of this condition usually requires surgical intervention. However, only a small number of patients are treated conservatively.

**Objective:**

The objective was to review the literature to determine the feasibility of conservative management for small bowel obstruction.

**Methods:**

A systematic search of the PubMed and Embase databases was performed using the keywords [small bowel obstruction AND pregnancy]. All original articles were then reviewed and included in this review if deemed suitable.

**Conclusion:**

Conservative management of small bowel obstruction in pregnant women is feasible if the patient is clinically stable and after ruling out bowel ischaemia and closed-loop obstruction.

**Supplementary Information:**

The online version contains supplementary material available at 10.1186/s13017-024-00541-y.

## Background

Intestinal obstruction is the third most common cause of acute abdomen during pregnancy. The incidence of small bowel obstruction (SBIO) during pregnancy varies from 1 in 66,431 to 1 in 1,500 deliveries [1]. 60% of SBIO cases are secondary to adhesions from prior surgery [2]. SBIO can be difficult to diagnose, as abdominal pain and vomiting in pregnant patients can be mistaken for labour pain or hyperemesis gravidarum [3]. 

Houston reported the first case of intestinal obstruction during pregnancy in 1830 [4], whereas Ludwig described the earliest case series in 1913 [5], where he reviewed a series of 95 cases of intestinal obstruction that occurred during pregnancy. As more abdominal operations were performed, general surgeons were more likely to encounter SBIO in pregnant women.

Initial reports strongly recommend exploratory surgery as standard treatment once intestinal obstruction is diagnosed to prevent further morbidity and mortality in both the mother and foetus [6]. Delivery of the baby in the same setting is sometimes needed if the surgical emergency threatens the pregnancy. This is, however, not ideal if the pregnancy is not at term. In recent years, some cases have shown that SBIO can be treated with conservative management [7, 8]. 

In this systematic review, we aimed to review the literature on SBIO in pregnant women to determine the feasibility of conservative management for this condition and which patients should be offered early surgical intervention.

## Methods

### Search strategy

A systematic search in the PubMed and Embase databases (including MEDLINE) was performed for studies about SBIO during pregnancy using the keywords [small bowel obstruction AND pregnancy]. This was performed by the first author (LXS) in January 2023.

### Review and study selection process

**Fig. 1:**
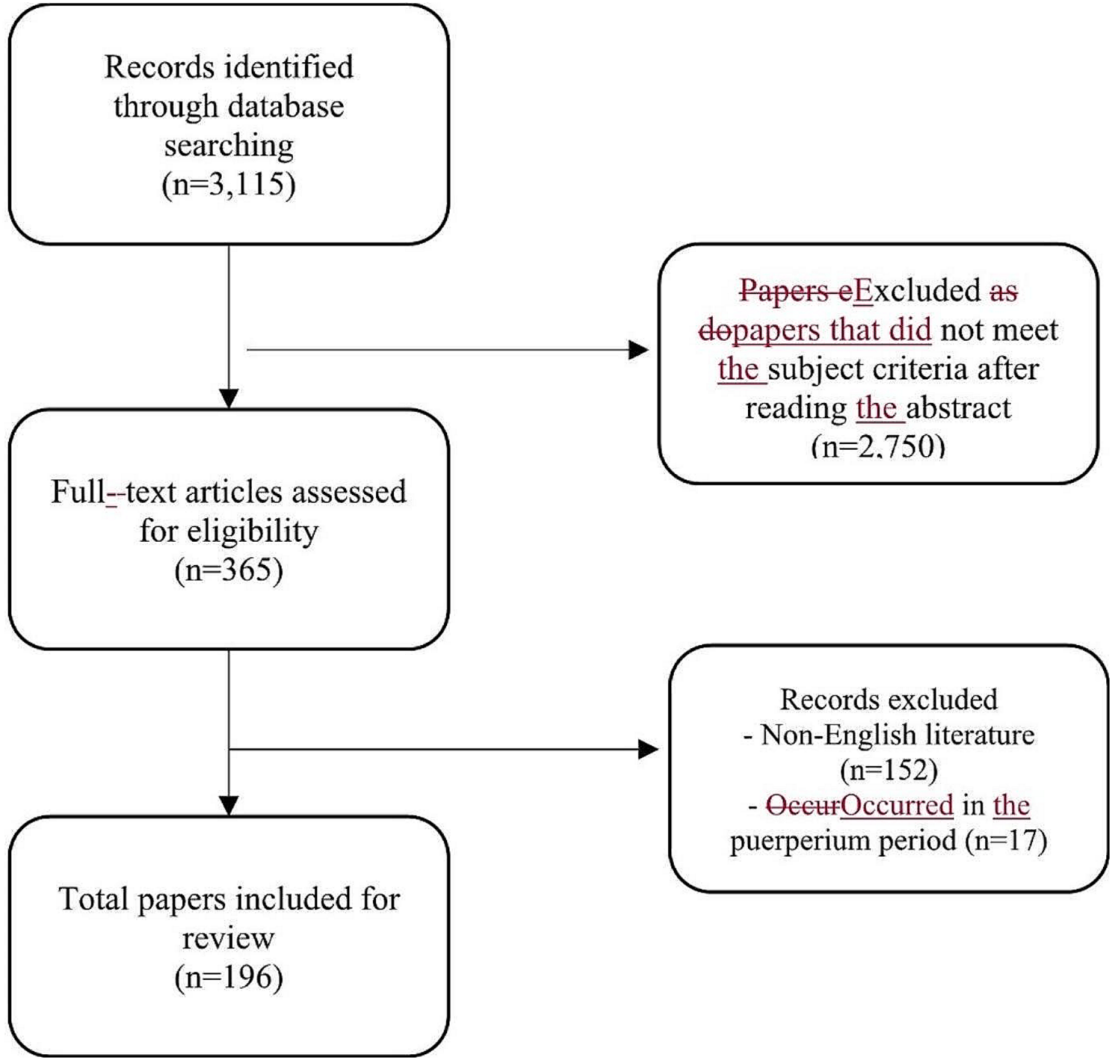
Search strategy

Titles and abstracts identified during the database search were assessed by two independent reviewers (LXS, BTWC) for potential eligibility. All original research articles, including case reports and series, were included. Articles were excluded according to the following exclusion criteria: articles that were not in published in English, articles on different subjects, conference proceedings, and animal studies. Disagreements between the two reviewers were settled through consensus. Articles deemed eligible for inclusion were obtained for full-text review and were assessed by two independent reviewers (LXS, BTWC). The reference lists of the included articles were searched for relevant papers that were not captured by the electronic search. A full diagram of the search strategy is provided in Fig. 1. A full list of included papers reviewed can be found in Appendix 1.

### Data extraction

The reviewers extracted and tabulated data from the eligible articles in a standardized form. Differences in the data were resolved through consensus. For each study, the following data were extracted: (1) age and gestational age of the patient; (2) presenting symptoms; (3) history of prior abdominal surgery; (4) imaging modality used to diagnose SBIO; (5) the cause of SBIO; (6) the management of SBIO (conservative treatment/failed conservative treatment/surgical intervention); (7) the time to definitive surgery (if the patient was offered surgical intervention); (8) the reason for offering surgical intervention; (9) the type of surgery performed (open vs. minimally-invasive surgery); (10) the surgical procedure performed; and 11) maternal and foetal mortality rates.

## Results

In the current review of the literature published from 1920 to January 2023, a total of 196 papers were included. These papers included case reports and case series of SBIO during pregnancy. We analysed 216 patients and their relevant outcomes.

Table 1 shows the demographics of pregnant patients upon presentation for SBIO. One patient presented twice during pregnancy for SBIO in the Gudbrand et al. series; [9] thus, the count was 217. The patients were further divided according to their gestational age and corresponding trimester of pregnancy. Perdue et al. states that obstruction due to adhesions are most common later in pregnancy [6]. This is also shown in our review, where 52.1% of patients presented in their third trimester.

Surgical history, particularly previous abdominal surgeries, was recorded and is shown in Table 1. We included patients with a history of previous Roux-En-Y Gastric Bypass (RYGB) in a separate entity.

**Table 1:** Demographics of patients at presentation

Gestational age at presentation	Number of patients (%)
First trimester	13 (5.9)
Second trimester (12 + 0 w onwards)	91 (42.0)
Third trimester (28 + 0 w onwards)	113 (52.1)
**Previous surgery**	
No previous abdominal surgery	68 (31.3)
Previous abdominal surgery	80 (36.9)
Previous bariatric surgery (Roux-En-Y Gastric Bypass)	67 (30.9)
Not mentioned	2 (0.9)

Table 2 shows the distribution of presenting complaints of patients and underlying causes of SBIO. In the analysing patients’ presenting complaints that prompted practitioners to suspect SBIO, the most common complaint was abdominal pain (54.7%), followed by vomiting (39.2%). As this review only analysed patients with SBIO, it is unsurprising that the patients rarely complained of obstipation, a typical feature of large bowel obstruction.

**Table 2:** Presenting complaints and underlying causes of SBIO in patients

Presenting complaints at initial presentation	Number of patients (%)
Abdominal pain	205 (54.7)
Vomiting	147 (39.2)
Obstipation	23 (6.1)
**Method of diagnosis**	
Clinical examination/intra-operative	67 (30.9)
Abdominal X-ray	43 (19.8)
Abdominal Ultrasound	24 (11.1)
Computed Tomography (CT) scan	49 (22.6)
Magnetic Resonance Imaging (MRI)	31 (14.3)
Endoscopic evaluation (OGD)	3 (1.3)
**Cause of SBIO**	
Adhesions	72 (33.3)
Internal herniation	63 (29.0)
Small bowel volvulus	30 (13.8)
Small bowel intussusception	26 (12.0)
Extrinsic compression of small bowel	4 (1.8)
Malrotation of small bowel	7 (3.2)
Negative findings on diagnostic laparoscopy	5 (2.3)
Other underlying cause	10 (4.6)

The diagnostic methods to diagnose SBIO vary concerning technological advances and regional differences. Before 1955, practitioners tended to diagnose SBIO by medical history or examination. In regions with better access to health care resources, practitioners typically use imaging, such as X-rays, computed tomography (CT) scans or magnetic resonance imaging (MRI) scans, to diagnose SBIO.

The most common cause of SBIO is adhesions (32.9%). The second most common cause is internal herniation; however, previous reviews have suggested that the second most common cause is volvulus [6]. This is likely due to the increasing popularity of bariatric surgery, especially RYGB, in recent years. The negative findings of 5 patients were noted on diagnostic laparoscopy. Gudbrand et al. offered diagnostic laparoscopy to rule out internal herniation in pregnant patients with previous RYGB who presented with SBIO [9]. In our review, extrinsic compression of the small bowel arose from the uterus (2 cases, in which 1 was due to compression of the bicornuate uterus and 1 in 2016 due to abdominal pregnancy [10]) fibroids (1 case), and recurrent malignant liposarcoma (1 case). Other underlying causes of SBIO included Crohn’s disease (2 cases), Meckel’s diverticulum as the lead point (3 cases), incarcerated incisional hernia (1 case), recurrent ventral hernia (1 case), phytobezoars (1 case), venous thrombosis (1 case) and the use of ondansetron (1 case).

Table 3 shows data on the management of SBIO. Most patients (92.2%) underwent surgery, with 22 patients having failed conservative management and ultimately requiring surgery. Out of the 39 patients who had conservative management, only 8 were started on total parenteral nutrition (TPN).

Most patients underwent surgery within 24 h. Given the high morbidity and mortality rates of acute abdominal emergencies in pregnant women, offering operative intervention in this population is less difficult. In this review, some patients were transferred from rural hospitals to a tertiary centre for definitive management of SBIO, resulting in a time to surgery of more than 1 day.

Nonresolving SBIO and clinical/scan/endoscopic findings were the common reasons for surgical intervention in our review (26.7%). Maternal distress was defined as nonreassuring vital signs such as fever, tachycardia, or hypotension. Clinical deterioration was defined as worsening symptoms such as pain or new clinical signs such as guarding or peritonism.

**Table 3:** Management of SBIO (conservative management vs. surgical intervention) and reasons for surgical intervention

Management of SBIO	Number of patients (%)
Conservative	16 (7.4)
Failed conservative (requiring surgical intervention)	22 (10.1)
Surgical intervention	179 (82.5)
**Use of TPN in patients treated conservatively**	
Yes	7 (18.4)
No	31 (81.6)
**The time interval between diagnosis and surgical intervention**	
Immediate (< 24 h)	124 (61.7)
Within 1 week	41 (20.4)
More than 1 week	15 (7.5)
Not mentioned	21 (10.4)
**Reasons for surgical intervention**	
Nonresolving condition	54 (26.9)
Clinical examination/scan/endoscopic findings	53 (26.4)
Clinical deterioration of the condition	40 (19.9)
Maternal distress	24 (11.9)
Foetal distress	12 (6.0)
Recurrent SBIO	5 (2.5)
Foetal demise	2 (0.9)
Not mentioned	11 (5.5)

Table 4 summarizes the method of operation analysed in our review and the types of procedures performed during the surgery. A total of 201 operations were performed in 217 patients. Of the 201 operations performed, laparotomy was the most common form of abdominal wall access (84.2%).

**Table 4:** Method of operation and operative procedures performed during surgical intervention

Method of operation	Number of patients (%)
Laparoscopic	32 (15.9)
Laparoscopic converted open	17 (8.5)
Laparotomy	152 (75.6)
**Operative procedures**	
Adhesiolysis	49 (20.3)
With bowel resection	83 (34.4)
With a concurrent caesarean section	56 (23.2)
With the closure of the internal defect	48 (20.0)
With Ladd’s procedure for malrotation	4 (1.7)
With resection of the tumour	1 (0.4)

This review included 216 pregnant women and 225 foetuses, comprising 7 pairs of twins and 1 set of triplets. Table 5 showed the survival rates of both patients and foetuses. Most of the patients and foetuses survived until term. A total of 3.7% of the mothers and 15.9% of the foetuses unfortunately did not survive.

**Table 5:** Maternal and foetal survival

Maternal survival	Number of patients (%)
yes	208 (96.3)
no	8 (3.7)
**Foetal survival**	
yes	189 (84.0)
no	36 (16.0)

## Discussion

Adhesions are the most common cause of SBIO in pregnant women [6]. The most frequent causes of postoperative adhesions in the general population are appendectomies and gynaecologic procedures [11]. 

Even without prior abdominal surgeries, adhesions cannot be ruled out as the underlying cause of SBIO, as 11% of adhesions are congenital [11]. 

The second most common cause of SBIO in our review was internal herniation (28.8%), mainly in patients with previous RYGB. This bariatric procedure, as a treatment for severe obesity, has increased in the past decade; most patients are women [12]. While the weight loss experienced by patients who undergo RYGB helps to reduce health risks, these patients are at risk of bariatric surgical complications, such as internal herniation, intussusception, and small bowel obstruction during pregnancy, due to the increase in intra-abdominal pressure caused by the gravid uterus and the reduction in excessive fat [13]. The American College of Obstetricians and Gynecologists (ACOG) published a review and practice bulletin in 2009 to inform obstetricians about SBIO in pregnant women as a well-recognized life-threatening late complication of RYGB [14]. Some authors have suggested that a bariatric surgeon should evaluate any pregnant patient with RYGB and abdominal complaints [15]. Some centres advocate a low threshold for diagnostic laparoscopy to rule out internal herniation. Internal hernias post-RYGB usually occur at 3 locations: (1) between the Roux limb mesentery and transverse mesentery (Petersen’s space); (2) at the defect in the transverse mesocolon; and (3) at the jejunojejunostomy mesenteric defect. All potential defects causing internal herniation must be inspected and closed during surgical exploration to prevent recurrence.

Abdominal pain is present in more than 85% of pregnant women with SBIO [16]. Due to the nature of pregnancy, abdominal pain can be confused with gastroenteritis or premature labour, whereas vomiting might be treated as hyperemesis gravidarum. Constant and nonremitting abdominal pain should alert practitioners to rule out the possibility of intestinal obstruction, particularly closed-loop obstructions, as the abdominal pain caused by gastroenteritis or uterine contractions is usually associated with periods of remission. Nausea and vomiting persisting into or starting in the third trimester should warrant further investigations to rule out intestinal obstruction.

Physical examination in pregnant women can be challenging and nonspecific due to the gravid uterus. The enlarged gravid uterus might mask abdominal distension caused by SBIO. Intermittent colicky pain caused by the obstructed bowel might be misinterpreted as labour pain. As the obstruction progresses, the uterus may contract due to the underlying irritation. This might confuse obstetricians, who incorrectly diagnose SBIO as early labour with contraction pain. Other clinical signs, such as fever, tachypnoea, hypotension, and tachycardia, usually appear later as secondary manifestations of severe acidosis and infection. Unfortunately, at this stage, this usually means that the bowel is compromised, and it might be too late to offer surgical intervention [17]. All these challenges highlight the clinical difficulties in diagnosing SBIO in pregnant women.

Laboratory tests yield little information besides electrolyte imbalances and impaired renal function in dehydrated patients. Leucocytosis is common during pregnancy, especially in late pregnancy and during labour. However, an increasing trend of leucocytosis over several hours is significant in gravid patients with suspected obstruction and should alert obstetricians to consider other causes [16]. 

Radiological imaging, such as X-rays of the abdomen, is used to aid in diagnosing SBIO. Diagnosis may be delayed due to apprehension about using X-ray imaging and exposing the foetus to radiation. However, significant maternal and foetal mortalities associated with acute abdominal emergencies outweigh the potential risk of radiation exposure to the foetus [10]. Sometimes, in the early stage of obstruction, a single film might not be sufficient to diagnose SBIO [18]. However, progressive bowel dilatation or air-fluid levels in serial films obtained at 4–6 h intervals are indicative of SBIO [19]. Previous literature described using contrast studies such as gastrografin or barium studies to diagnose SBIO [17]. This has been gradually phased out in recent years, especially with the widespread availability of advanced imaging modalities such as CT and MRI scans.

The use of ultrasound is widespread, as this modality does not confer any radiation exposure to the foetus. However, one study reported that only 55% of patients had ultrasound findings similar to the surgical findings [20]. Thus, ultrasound is not the most sensitive modality to rule out SBIO in pregnant patients.

CT scans play a role as a diagnostic modality in the general population. Abdominal CT scan with oral and intravenous contrast is the best radiological tool to evaluate patients with previous RYGB who present with obstructive symptoms suggestive of internal hernias [21]. Unfortunately, radiation exposure to the foetus is highest when a full scan of the abdomen and pelvis is performed [22]. Hence, the benefits of a CT scan should be weighed against the cumulative radiation exposure to the foetus. These levels vary by institution; practitioners should be aware of the cumulative radiation exposure to the foetus. MRI has been gaining popularity in pregnant women to diagnose intestinal obstruction and the underlying cause, as this modality provides excellent soft tissue multiplanar imaging without ionizing radiation [23]. The use of gadolinium remains contraindicated as this agent crosses the placenta, and the effects on the foetus are not fully understood. With the increasing use of MRI and CT scans, early diagnosis of SBIO in pregnant women is possible, especially in regions where health care resources are more accessible.

With an earlier diagnosis, health care practitioners can intervene early to reduce maternal and foetal morbidity and mortality. In this review, most patients (61%) underwent surgery in the first 24 h after presentation. Due to the higher stakes in pregnant patients, past literature strongly recommended operative intervention for intestinal obstruction in pregnant women [17]. As early as 1932, Murray Blair suggested that the abdomen should be opened, and the cause should be ascertained when intestinal obstruction occurs during a normal intrauterine pregnancy [24]. Harper WB Jr states that conservative treatment for intestinal obstruction during pregnancy is generally not recommended because of the frequency of closed-loop obstruction, which occurs in up to 40% of patients [25]. Perdue et al. reported a significant risk in treating SBIO during pregnancy with tube decompression alone, except possibly in pregnant patients with sigmoid volvulus [6]. 

Pregnant women with RYGB-associated SBIO generally require surgical exploration for diagnosis and treatment, even if the condition responds to conservative treatment measures [26]. This is because the common causes of SBIO post-RYGB, such as adhesions, mesenteric defects, and stenosis, persist without surgical intervention, potentially leaving the patient at risk for recurrent and catastrophic SBIO. Gudbrand et al. described a case of recurrent SBIO caused by internal herniation in a pregnant woman with previous RYGB despite the closure of Petersen’s defect in the first surgery performed laparoscopically. She eventually underwent laparotomy and closure of the Petersen defect when the condition recurred within 8 weeks [9]. In this review, all patients with SBIO and a history of previous RYGB were operated on. All mothers and babies survived except in four cases, including two cases of foetal mortality, one case of maternal mortality, and one case in which both the mother and baby did not survive due to a delay in diagnosis.

Surgical management and techniques in this group of patients do not differ from those in the general population. Once the decision for surgery is made, the consideration of which surgical approach to utilize (laparoscopy versus laparotomy) is based on the surgeon’s skills and the availability of the appropriate staff and equipment. With the increasing popularity of minimally invasive surgery (MIS), there are concerns about the use of MIS in pregnant patients due to the risk to the foetus from trocar insertion and CO_2_ insufflation, the risk to the mother from pneumoperitoneum causing a reduced venous return to the heart and the ability to obtain adequate view with a gravid uterus. It has been shown that laparoscopy can be performed safely during any trimester of pregnancy with minimal morbidity to the foetus and mother [23]. Nezhat FR et al. reported favourable outcomes in 51 cases of abdominal operative laparoscopy performed in pregnant patients [27]. In our review, 15.9% of patients underwent successful MIS. Most patients still undergo laparotomy, especially if the case is complicated or the diagnosis is uncertain.

Management of the foetus at the time of operation will depend on the gestational age of the foetus as well as the maternal condition at the time of laparotomy. Unless the pregnancy is at term and the foetus is ready to be delivered, surgeons should minimize manipulation of the uterus as much as possible. Hypotension and hypoxia, while the mother is anaesthetized, are the two most common causes of foetal death or abortion [28]. In this review, only 21% of foetuses were delivered during surgery. If foetal delivery is needed during the operation, this should precede relief of the obstruction.

Conservative management of SBIO in pregnant women was frowned upon, as later intervention often leads to dire consequences. Chiedozi LC et al. reported a case where a pregnant woman with SBIO was started on a trial of conservative treatment [29]. Ten days later, the patient experienced maternal collapse from shock. She was resuscitated and brought to the operating theatre immediately. Most of the intestine was found in a tight volvulus at laparotomy. Eight feet of the gangrenous small intestine were resected, but both the patient and foetus died 12 h after the operation. This case highlights the importance of carefully selecting patients for a trial of conservative treatment, especially if no prior imaging has been done to rule out life-threatening emergencies such as closed-loop obstruction or volvulus.

If closed-loop obstruction and bowel ischaemia have been ruled out and the patient has no prior history of RYGB, stable patients with reassuring foetal tracing can be started on a trial of conservative management. Most of this carefully selected group of patients have SBIO due to adhesions with no worrisome findings on CT or MRI. M Phillips et al. described a case of recurrent SBIO secondary to adhesions in a pregnant woman. She was placed on conservative management for 10 weeks with an elemental diet via tube feeding. She delivered a healthy baby boy via elective caesarean section due to breech presentation without any complications [30]. 

SBIO can cause malnutrition in pregnant women due to the failure to absorb nutrients from the gut. This will increase the risk of spontaneous abortion, congenital malformations, intrauterine growth restriction (IUGR), preterm delivery, and perinatal mortality and morbidity [31]. For the past decades, the use of TPN was described as providing adequate nutritional support for malnourished pregnant women. Caruso et al. demonstrated that intravenous nutrition is well tolerated and can be administered safely and effectively to malnourished pregnant women without catheter-related or metabolic complications [32]. After appropriate counselling, patients who require TPN during conservative management for SBIO should be referred to experienced obstetric centres where TPN and careful monitoring of the maternal and foetal conditions can be carried out. In an unfortunate case, Lee S et al. reported a case where the patient presented with SBIO secondary to adhesions in the second trimester [33]. The patient was started on TPN and observed closely for 18 days. Unfortunately, the foetus developed acute onset foetal brain haemorrhage, likely secondary to vitamin K deficiency, and thus surgical intervention had to be offered. The baby died shortly after delivery via caesarean section in the same setting. This highlights the need for close observation of both maternal and foetal conditions. Due to the high costs of TPN and limited availability, especially in areas with limited access to health care resources, TPN initiation in pregnant patients with SBIO who are treated conservatively can be difficult.

Over the years, there has been an increase in the survival of pregnant patients with SBIO. A 75% maternal fatality rate was reported in Ludwig’s series in 1913 [5]. Subsequently, Harper WB Jr reported a maternal mortality rate of 22% from 1928 to 58, [25], and Morris reported a maternal mortality rate of 12% in 1965 [16]. Immediate causes of maternal death include irreversible shock and infection. The overall maternal mortality rate in our series was 3.7%. This improvement is likely due to early diagnosis and early intervention.

Compared to the maternal mortality rate, the foetal mortality rate is usually higher. Perdue et al. reported a foetal mortality rate of 26% compared to a maternal mortality rate of 6% in his case series. In our series, the foetal mortality is 16%, which is lower than what was reported by Perdue et al. in 1992. This is a reflect of medical improvement over the years, where early diagnosis and intervention can be carried out promptly.

As the mother’s condition deteriorates, the foetal condition will worsen rapidly. There was a dramatic progression of foetal mortality as patients approached the third trimester, with 15 foetal deaths occurring in the third trimester group. It is tragic for patients to lose their viable babies due to abdominal conditions. In our review, there were 5 cases of maternal and foetal mortality. Four out of the 5 patients were operated on immediately upon presentation. Despite immediate surgical intervention, the damage caused by profound shock was irreversible, resulting in the demise of the patients and the foetuses. One can only hope that pregnant patients have better access to health care and thus present earlier when they are ill.

## Conclusion

SBIO during pregnancy is a relatively rare condition with dire consequences if missed. With medical advancements, there should be no reason for missed diagnosis, and early intervention should be provided to prevent maternal and foetal mortality and morbidity. After diagnosing the underlying cause of SBIO and ruling out bowel ischaemia or closed-loop obstruction, it is worth attempting conservative management to avoid morbidity from surgical intervention. This is best done with appropriate close monitoring of both the mother and foetus, considering the commencement of TPN to prevent complications of malnourishment until the SBIO is resolved.

### Electronic supplementary material

Below is the link to the electronic supplementary material.

Supplementary Material 1

## Data Availability

Not applicable.

## Abbreviations

SBIO: Small Bowel Intestinal Obstruction
RYGB: Roux-En-Y Gastric Bypass
CT: Computed Tomography
MRI: Magnetic Resonance Imaging
TPN: Total Parenteral Nutrition
ACOG: American College of Obstetricians and Gynecologists
MIS: Minimally Invasive Surgery
IUGR: Intrauterine Growth Restriction
