# Supplementary material for: Can small bowel obstruction during pregnancy be treated with conservative management? A review
Source: World J Emerg Surg. 2024 Apr 10;19:13. doi: 10.1186/s13017-024-00541-y (PMC11007914; doi:10.1186/s13017-024-00541-y)
Supplement: Supplementary file 1 — Supplementary Material 1 [file 13017_2024_541_MOESM1_ESM.docx]

Appendix 1. Full Article List

**Pubmed**

1. Jackson CE. ACUTE INTESTINAL OBSTRUCTION DUE TO PREGNANCY IN A BICORNUATE UTERUS. Br Med J. 1920 Feb 7;1(3084):185. doi: 10.1136/bmj.1.3084.185. PMID: 20769785; PMCID: PMC2337177.
2. JAMES DW. Intestinal obstruction during late pregnancy. Br Med J. 1946 Mar 30;1:485. PMID: 20988605.
3. CLARK CW, MITCHELL R, MITCHELL JR. Pregnancy complicated by acute intestinal obstruction. Can Med Assoc J. 1952 May;66(5):475-6. PMID: 14925859; PMCID: PMC1822491.
4. HARER WB Jr, HARER WB Sr. Volvulus complicating pregnancy and puerperium; report of three cases and review of literature. Obstet Gynecol. 1958 Oct;12(4):399-406. PMID: 13590653.
5. HARPER WB Jr. Intestinal obstruction associated with pregnancy. Report of ten cases. Obstet Gynecol. 1962 Jan;19:11-5. PMID: 13904786.
6. Moore DT, Watts CD, Wilbanks GD. Intussusception complicating pregnancy. Report of a case. J Natl Med Assoc. 1967 Jan;59(1):20-1. PMID: 6038581; PMCID: PMC2611281.
7. Hammouda AA. Acute intestinal obstruction during pregnancy: a brief review and report of two cases. Aust N Z J Obstet Gynaecol. 1967 May;7(2):101-3. doi: 10.1111/j.1479-828x.1967.tb02498.x. PMID: 5233015.
8. Goodwin DP. Volvulus of the small intestine in labour. Br J Surg. 1968 Jun;55(6):469-70. doi: 10.1002/bjs.1800550617. PMID: 5651550.
9. Perry RA, Carroll KJ. Labour complicated by intestinal volvulus. Aust N Z J Obstet Gynaecol. 1968 May;8(2):87-8. doi: 10.1111/j.1479-828x.1968.tb00689.x. PMID: 5242150.
10. Weston PV, Lindheimer MD. Intermittent intestinal obstruction simulating hyperemesis gravidarum. Obstet Gynecol. 1971 Jan;37(1):106-8. PMID: 5538904.
11. Beck WW Jr. Intestinal obstruction in pregnancy. Obstet Gynecol. 1974 Mar;43(3):374-8. PMID: 4814455.
12. Hill LM, Symmonds RE. Small bowel obstruction in pregnancy. A review and report of four cases. Obstet Gynecol. 1977 Feb;49(2):170-3. PMID: 834399.
13. Holland GA. Unusual case of intestinal obstruction in late pregnancy. Can Med Assoc J. 1978 Jun 24;118(12):1488, 1493. PMID: 657046; PMCID: PMC1818101.
14. Sivasuriya M, Wickramasinghe SY. Volvulus of the small bowel complicating mid-trimester pregnancy. Aust N Z J Obstet Gynaecol. 1979 Nov;19(4):247-8. doi: 10.1111/j.1479-828x.1979.tb01386.x. PMID: 295647.
15. Cox KL, Byrne WJ, Ament ME. Home total parenteral nutrition during pregnancy: a case report. JPEN J Parenter Enteral Nutr. 1981 May-Jun;5(3):246-9. doi: 10.1177/0148607181005003246. PMID: 6788976.
16. Graubard Z, Graham KM, Schein M. Small-bowel obstruction in pregnancy after Scopinaro weight reduction operation. A case report. S Afr Med J. 1988 Jan 23;73(2):127-8. PMID: 3340918.
17. Adedeji O, McAdam WA. Intussusception in ileostomy in a pregnant woman. Postgrad Med J. 1992 Jan;68(795):67-8. doi: 10.1136/pgmj.68.795.67-a. PMID: 1561200; PMCID: PMC2399302.
18. Liddicoat AJ, Lloyd DC. Case report: small bowel volvulus presenting during pregnancy. Clin Radiol. 1992 Oct;46(4):286-7. doi: 10.1016/s0009-9260(05)80174-6. PMID: 1424456.
19. Wax JR, Christie TL. Complete small-bowel volvulus complicating the second trimester. Obstet Gynecol. 1993 Oct;82(4 Pt 2 Suppl):689-91. PMID: 8378014.
20. Matthews CM, Soper DE. Midgut volvulus associated with pregnancy. South Med J. 1993 Jul;86(7):819-20. doi: 10.1097/00007611-199307000-00021. PMID: 8322092.
21. Meyerson S, Holtz T, Ehrinpreis M, Dhar R. Small bowel obstruction in pregnancy. Am J Gastroenterol. 1995 Feb;90(2):299-302. PMID: 7847305.
22. Walker M, Sylvain J, Stern H. Bowel obstruction in a pregnant patient with ileal pouch-anal anastomosis. Can J Surg. 1997 Dec;40(6):471-3. PMID: 9416260; PMCID: PMC3950045.
23. Damore LJ 2nd, Damore TH, Longo WE, Miller TA. Congenital intestinal malrotation causing gestational intestinal obstruction. A case report. J Reprod Med. 1997 Dec;42(12):805-8. PMID: 9437596.
24. Ventura-Braswell AM, Satin AJ, Higby K. Delayed diagnosis of bowel infarction secondary to maternal midgut volvulus at term. Obstet Gynecol. 1998 May;91(5 Pt 2):808-10. doi: 10.1016/s0029-7844(97)00712-6. PMID: 9572169.
25. Chiedozi LC, Ajabor LN, Iweze FI. Small intestinal obstruction in pregnancy and puerperium. Saudi J Gastroenterol. 1999 Sep;5(3):134-9. PMID: 19864739.
26. Aggarwal N, Sawhney H, Vasishta K, Anju. Small bowel volvulus complicating pregnancy. Aust N Z J Obstet Gynaecol. 1999 May;39(2):254-5. doi: 10.1111/j.1479-828x.1999.tb03386.x. PMID: 10755793.
27. Watanabe S, Otsubo Y, Shinagawa T, Araki T. Small bowel obstruction in early pregnancy treated by jejunotomy and total parenteral nutrition. Obstet Gynecol. 2000 Nov;96(5 Pt 2):812-3. doi: 10.1016/s0029-7844(00)01052-8. PMID: 11094216.
28. Yahchouchy E, Zaarour P, Prove S, Fingerhut A. Recurrent idiopathic small bowel volvulus during pregnancy. ANZ J Surg. 2001 Mar;71(3):193-4. doi: 10.1046/j.1440-1622.2001.02065.x. PMID: 11277153.
29. Jones I, Hillen J. Small bowel obstruction during pregnancy: a case report. Aust N Z J Obstet Gynaecol. 2002 Aug;42(3):311-2. doi: 10.1111/j.0004-8666.2002.300_7.x. PMID: 12230075.
30. Dilbaz S, Gelisen O, Caliskan E, Caliskan S, Gokcin H, Haberal A. Small bowel volvulus in pregnancy. Eur J Obstet Gynecol Reprod Biol. 2003 Dec 10;111(2):204-6. doi: 10.1016/s0301-2115(03)00203-3. PMID: 14597252.
31. Girgis MM, Malas HZ, Ahmad I, Obaiden AK. Intussusception complicating triplet pregnancy. Saudi Med J. 2004 Apr;25(4):515-7. PMID: 15083228.
32. Phillips M, Curtis P, Karanjia N. An elemental diet for bowel obstruction in pregnancy: a case study. J Hum Nutr Diet. 2004 Dec;17(6):543-5. doi: 10.1111/j.1365-277X.2004.00565.x. PMID: 15546432.
33. Baker MT, Kothari SN. Successful surgical treatment of a pregnancy-induced Petersen's hernia after laparoscopic gastric bypass. Surg Obes Relat Dis. 2005 Sep-Oct;1(5):506-8. doi: 10.1016/j.soard.2005.07.008. PMID: 16925279.
34. Kakarla N, Dailey C, Marino T, Shikora SA, Chelmow D. Pregnancy after gastric bypass surgery and internal hernia formation. Obstet Gynecol. 2005 May;105(5 Pt 2):1195-8. doi: 10.1097/01.AOG.0000152352.58688.27. PMID: 15863579.
35. Peters CW, Layon AJ, Edwards RK. Cardiac arrest during pregnancy. J Clin Anesth. 2005 May;17(3):229-34. doi: 10.1016/j.jclinane.2004.09.003. PMID: 15986554.
36. Rudloff U, Jobanputra S, Smith-Levitin M, Kessler E. Meckel's diverticulum complicating pregnancy. Case report and review of the literature. Arch Gynecol Obstet. 2005 Jan;271(1):89-93. doi: 10.1007/s00404-004-0641-y. Epub 2004 Jul 28. PMID: 15290167.
37. Charles A, Domingo S, Goldfadden A, Fader J, Lampmann R, Mazzeo R. Small bowel ischemia after Roux-en-Y gastric bypass complicated by pregnancy: a case report. Am Surg. 2005 Mar;71(3):231-4. PMID: 15869139.
38. Choi SA, Park SJ, Lee HK, Yi BH, Kim HC. Preoperative diagnosis of small-bowel intussusception in pregnancy with the use of sonography. J Ultrasound Med. 2005 Nov;24(11):1575-7. doi: 10.7863/jum.2005.24.11.1575. PMID: 16239665.
39. Bellanger DE, Ruiz JF, Solar K. Small bowel obstruction complicating pregnancy after laparoscopic gastric bypass. Surg Obes Relat Dis. 2006 Jul-Aug;2(4):490-2. doi: 10.1016/j.soard.2006.04.002. PMID: 16925388.
40. Baykal C, Al A, Ozer S, Hizli D, Gökçin H. Ileal resection for gangrenous ileal volvulus in a term pregnancy: a case report. Arch Gynecol Obstet. 2006 Feb;273(5):304-6. doi: 10.1007/s00404-005-0058-2. Epub 2005 Dec 1. PMID: 16320064.
41. Chang YT, Huang YS, Chan HM, Huang CJ, Hsieh JS, Huang TJ. Intestinal obstruction during pregnancy. Kaohsiung J Med Sci. 2006 Jan;22(1):20-3. doi: 10.1016/S1607-551X(09)70215-3. PMID: 16570564.
42. Ahmed AR, O'Malley W. Internal hernia with Roux loop obstruction during pregnancy after gastric bypass surgery. Obes Surg. 2006 Sep;16(9):1246-8. doi: 10.1381/096089206778392310. PMID: 16989713.
43. Wang CB, Hsieh CC, Chen CH, Lin YH, Lee CY, Tseng CJ. Strangulation of upper jejunum in subsequent pregnancy following gastric bypass surgery. Taiwan J Obstet Gynecol. 2007 Sep;46(3):267-71. doi: 10.1016/S1028-4559(08)60032-7. PMID: 17962108.
44. Redlich A, Rickes S, Costa SD, Wolff S. Small bowel obstruction in pregnancy. Arch Gynecol Obstet. 2007 May;275(5):381-3. doi: 10.1007/s00404-006-0262-8. Epub 2006 Oct 18. PMID: 17047974.
45. Ou KY, Lee YM, Shen CR, Chen HS, Tsai EM. Volvulus in pregnancy: a diagnostic dilemma. Kaohsiung J Med Sci. 2007 Mar;23(3):147-50. doi: 10.1016/S1607-551X(09)70390-0. PMID: 17389180.
46. Wax JR, Wolff R, Cobean R, Pinette MG, Blackstone J, Cartin A. Intussusception complicating pregnancy following laparoscopic Roux-en-Y gastric bypass. Obes Surg. 2007 Jul;17(7):977-9. doi: 10.1007/s11695-007-9153-8. PMID: 17894160.
47. Essilfie P, Hussain M, Stokes IM. Small bowel infarction secondary to volvulus during pregnancy: a case report. J Reprod Med. 2007 Jun;52(6):553-4. PMID: 17694982.
48. Gould CH, Maybee GJ, Leininger B, Winter WE 3rd. Primary intussusception in pregnancy: a case report. J Reprod Med. 2008 Sep;53(9):703-7. PMID: 18839827.
49. Pelikan H, Stoot J, Meens-Koreman S, Teijink J. Congenital intestinal malrotation masquerading as hyperemesis gravidarum. Int J Gynaecol Obstet. 2008 Jul;102(1):74-5. doi: 10.1016/j.ijgo.2008.01.014. Epub 2008 Mar 4. PMID: 18313058.
50. Torres-Villalobos GM, Kellogg TA, Leslie DB, Antanavicius G, Andrade RS, Slusarek B, Prosen TL, Ikramuddin S. Small bowel obstruction and internal hernias during pregnancy after gastric bypass surgery. Obes Surg. 2009 Jul;19(7):944-50. doi: 10.1007/s11695-008-9681-x. Epub 2008 Oct 2. PMID: 18830790.
51. Tohamy AE, Eid GM. Laparoscopic reduction of small bowel intussusception in a 33-week pregnant gastric bypass patient: surgical technique and review of literature. Surg Obes Relat Dis. 2009 Jan-Feb;5(1):111-5. doi: 10.1016/j.soard.2008.09.008. Epub 2008 Sep 18. PMID: 19161938.
52. Young BC, Fugelso D, Takoudes T. Incisional hernia with bowel incarceration and obstruction at 34 weeks gestational age. Arch Gynecol Obstet. 2009 Jun;279(6):905-7. doi: 10.1007/s00404-008-0810-5. Epub 2008 Oct 11. PMID: 18850105.
53. Luhmann A, Tait R, Hassn A. Intussusception in a 20 weeks pregnant woman: a case report. Cases J. 2009 Aug 5;2:6546. doi: 10.4076/1757-1626-2-6546. PMID: 19918530; PMCID: PMC2769300.
54. Zapardiel I, DelaFuente-Valero J, Herrero-Gamiz S, Sanfrutos L, Aguaron A, Bajo-Arenas J. Large Meckel's diverticulum complicating pregnancy with an intestinal obstruction. Acta Obstet Gynecol Scand. 2009;88(4):492-3. doi: 10.1080/00016340902741216. PMID: 19191079.
55. Scheuermeyer F. Precipitous decline of gravid patient with congenital small bowel malrotation. Am J Emerg Med. 2009 Jun;27(5):629.e5-6. doi: 10.1016/j.ajem.2008.08.034. PMID: 19497481.
56. Osime OC, Onakewhor J, Irowa OO. Intussusception in pregnancy--a rarely considered diagnosis. Afr J Reprod Health. 2010 Mar;14(1):145-8. PMID: 20695148.
57. Nishida Y. MR imaging of a maternal strangulated ileus during early pregnancy. Acta Obstet Gynecol Scand. 2010 Oct;89(10):1367-8. doi: 10.3109/00016349.2010.513430. PMID: 20846072.
58. Seow-En I, Seow-Choen F, Tseng PT. Prior uterine perforation resulting in intestinal obstruction in a subsequent pregnancy. Tech Coloproctol. 2010 Dec;14(4):369. doi: 10.1007/s10151-010-0637-5. Epub 2010 Aug 13. PMID: 20706758.
59. Witherspoon P, Chalmers AG, Sagar PM. Successful pregnancy after laparoscopic ileal pouch-anal anastomosis complicated by small bowel obstruction secondary to a single band adhesion. Colorectal Dis. 2010 May;12(5):490-1. doi: 10.1111/j.1463-1318.2009.01855.x. Epub 2009 Apr 2. PMID: 19341400.
60. Gaikwad A, Ghongade D, Kittad P. Fatal midgut volvulus: a rare cause of gestational intestinal obstruction. Abdom Imaging. 2010 Jun;35(3):288-90. doi: 10.1007/s00261-009-9519-6. Epub 2009 May 7. PMID: 19421807.
61. Gagné DJ, DeVoogd K, Rutkoski JD, Papasavas PK, Urbandt JE. Laparoscopic repair of internal hernia during pregnancy after Roux-en-Y gastric bypass. Surg Obes Relat Dis. 2010 Jan-Feb;6(1):88-92. doi: 10.1016/j.soard.2009.06.002. Epub 2009 Jun 18. PMID: 19733511.
62. Naef M, Mouton WG, Wagner HE. Small-bowel volvulus in late pregnancy due to internal hernia after laparoscopic Roux-en-Y gastric bypass. Obes Surg. 2010 Dec;20(12):1737-9. doi: 10.1007/s11695-009-9802-1. Epub 2009 Jan 28. PMID: 19184255.
63. Gazzalle A, Braun D, Cavazzola LT, Wendt LR, Navarini D, Fauri Mde A, Vitola SP. Late intestinal obstruction due to an intestinal volvulus in a pregnant patient with a previous Roux-en-Y gastric bypass. Obes Surg. 2010 Dec;20(12):1740-2. doi: 10.1007/s11695-009-9825-7. Epub 2009 Mar 25. PMID: 19319613.
64. Shui LH, Rafi J, Corder A, Mowbray D. Mid-gut volvulus and mesenteric vessel thrombosis in pregnancy: case report and literature review. Arch Gynecol Obstet. 2011 Mar;283 Suppl 1:39-43. doi: 10.1007/s00404-010-1789-2. Epub 2010 Dec 12. PMID: 21153648.
65. Lin ID, Wang HI, Wang PH, Huang CY, Chen CC, Shiu HL, Chiang MM, Lin HJ, Chen CY, Yang MJ, Chao KC. Mesentery band-like adhesion which caused entrapment of duodenum at 32 gestational weeks. Taiwan J Obstet Gynecol. 2011 Dec;50(4):534-6. doi: 10.1016/j.tjog.2011.10.026. PMID: 22212333.
66. Ekanem VJ, Umukoro DO, Igberase G. Intestinal obstruction due to bilateral ovarian cystic teratoma in a pregnant woman: report of a case. Afr J Reprod Health. 2011 Mar;15(1):117-20. PMID: 21987947.
67. Lin H, Lin CC, Huang WT. Idiopathic superior mesenteric vein thrombosis resulting in small bowel ischemia in a pregnant woman. Case Rep Obstet Gynecol. 2011;2011:687250. doi: 10.1155/2011/687250. Epub 2011 Sep 14. PMID: 22567515; PMCID: PMC3335606.
68. Vassiliou I, Tympa A, Derpapas M, Kottis G, Vlahos N. Small Bowel Ischemia due to Jejunum Volvulus in Pregnancy: A Case Report. Case Rep Obstet Gynecol. 2012;2012:485863. doi: 10.1155/2012/485863. Epub 2012 Dec 12. PMID: 23304583; PMCID: PMC3530760.
69. Spring A, Lee M, Patchett S, Deasy J, Wilson I, Cahill RA. Ileostomy obstruction in the third trimester of pregnancy. Colorectal Dis. 2012 Sep;14(9):e631-2. doi: 10.1111/j.1463-1318.2012.02972.x. PMID: 22498003.
70. Li Y, Ang M, Miller JA. A rare cause of bowel obstruction in pregnancy. J Surg Case Rep. 2012 Dec 19;2012(12):rjs034. doi: 10.1093/jscr/rjs034. PMID: 24968425; PMCID: PMC3855217.
71. Wasadikar PP, Harbade SR, Meshram PS, Wasadikar AP, Varudkar AS, Deshpande SS. Strangulating ileocolic intussusception in pregnancy. J Obstet Gynaecol India. 2012 Dec;62(Suppl 1):6-7. doi: 10.1007/s13224-013-0353-y. Epub 2013 Mar 12. PMID: 24293856; PMCID: PMC3632687.
72. Renault K, Gyrtrup HJ, Damgaard K, Hedegaard M, Sørensen JL. Pregnant woman with fatal complication after laparoscopic Roux-en-Y gastric bypass. Acta Obstet Gynecol Scand. 2012 Jul;91(7):873-5. doi: 10.1111/j.1600-0412.2012.01421.x. Epub 2012 May 22. PMID: 22524680.
73. Tuyeras G, Pappalardo E, Msika S. Acute small bowel obstruction following laparoscopic Roux-en-Y gastric bypass during pregnancy: two different presentations. J Surg Case Rep. 2012 Jul 1;2012(7):1. doi: 10.1093/jscr/2012.7.1. PMID: 24960726; PMCID: PMC3649561.
74. Borghede MK, Vinter-Jensen L, Andersen JC, Mortensen PB, Rasmussen HH. Reconstruction of short bowel syndrome after internal hernia in a pregnant woman with previous bariatric surgery. Int J Surg Case Rep. 2013;4(12):1100-3. doi: 10.1016/j.ijscr.2013.08.025. Epub 2013 Oct 8. PMID: 24240078; PMCID: PMC3860043.
75. Lazaridis A, Maclaran K, Behar N, Narayanan P. A rare case of small bowel obstruction secondary to ovarian torsion in an IVF pregnancy. BMJ Case Rep. 2013 Feb 15;2013:bcr2013008551. doi: 10.1136/bcr-2013-008551. PMID: 23417952; PMCID: PMC3603835.
76. Li M, Chen JF, Wei F. Double intussusceptions with small intestinal adenoma in pregnancy. Eur J Obstet Gynecol Reprod Biol. 2013 Nov;171(1):188-9. doi: 10.1016/j.ejogrb.2013.08.018. Epub 2013 Aug 16. PMID: 24021318.
77. Ranade A, Shah BC, Oleynikov D. Intussusception during pregnancy of a gastric bypass patient. Surg Obes Relat Dis. 2013 Sep-Oct;9(5):e84-5. doi: 10.1016/j.soard.2013.01.018. Epub 2013 Feb 6. PMID: 23477956.
78. Rauff S, Chang SK, Tan EK. Intestinal obstruction in pregnancy: a case report. Case Rep Obstet Gynecol. 2013;2013:564838. doi: 10.1155/2013/564838. Epub 2013 Feb 6. PMID: 23476843; PMCID: PMC3580938.
79. Deront Bourdin F, Iannelli A, Delotte J. Phytobezoar: an unexpected cause of bowel obstruction in a pregnant woman with a history of Roux-en-Y gastric bypass. Surg Obes Relat Dis. 2014 Nov-Dec;10(6):e49-51. doi: 10.1016/j.soard.2014.07.009. Epub 2014 Jul 19. PMID: 25443076.
80. Zachariah SK, Fenn MG. Acute intestinal obstruction complicating pregnancy: diagnosis and surgical management. BMJ Case Rep. 2014 Mar 6;2014:bcr2013203235. doi: 10.1136/bcr-2013-203235. PMID: 24604803; PMCID: PMC3948142.
81. Hwang SM, Na YS, Cho Y, You DG, Lee JJ. Midgut volvulus as a complication of intestinal malrotation in a term pregnancy. Korean J Anesthesiol. 2014 Dec;67(Suppl):S98-9. doi: 10.4097/kjae.2014.67.S.S98. PMID: 25598929; PMCID: PMC4296003.
82. Nameirakpam S, Keishing S, Laishram J, Devi S R. Small Intestine Ischaemia due to Volvulus during Pregnancy. J Clin Diagn Res. 2014 Apr;8(4):ND01-2. doi: 10.7860/JCDR/2014/7929.4242. Epub 2014 Apr 15. PMID: 24959475; PMCID: PMC4064923.
83. Serra AE, Fong A, Chung JH. A gut-wrenching feeling: pregnancy complicated by massive ventral hernia with bowel obstruction. Am J Obstet Gynecol. 2014 Jul;211(1):79.e1-2. doi: 10.1016/j.ajog.2014.03.006. Epub 2014 Mar 5. PMID: 24607750.
84. Cong Q, Li X, Ye X, Sun L, Jiang W, Han Z, Lu W, Xu H. Small bowel volvulus in mid and late pregnancy: can early diagnosis be established to avoid catastrophic outcomes? Int J Clin Exp Med. 2014 Nov 15;7(11):4538-43. PMID: 25550984; PMCID: PMC4276242.
85. Bokslag A, Jebbink J, De Wit L, Oudijk M, Ribbert L, Tahri S, van Pampus M. Intussusception during pregnancy after laparoscopic Roux-en-Y gastric bypass. BMJ Case Rep. 2014 Nov 18;2014:bcr2014205357. doi: 10.1136/bcr-2014-205357. Erratum in: BMJ Case Rep. 2015;2015. pii: bcr2014205357corr1. doi: 10.1136/bcr-2014-205357corr1. van Pampus, Mariëlle [corrected to van Pampus, Maria G]. PMID: 25406213; PMCID: PMC4244488.
86. Pearce P, Patel H, Vijithan T, Craddock CT, Banerjee S. Gestational intestinal obstruction complicated by cortical blindness - a multidisciplinary case report. JRSM Open. 2014 Jun 9;5(7):2054270414531120. doi: 10.1177/2054270414531120. PMID: 25057408; PMCID: PMC4100233.
87. Bosman WM, Veger HT, Hedeman Joosten PP, Ritchie ED. Ileocaecal intussusception due to submucosal lipoma in a pregnant woman. BMJ Case Rep. 2014 Feb 14;2014:bcr2013203110. doi: 10.1136/bcr-2013-203110. PMID: 24532237; PMCID: PMC3926411.
88. Wong TF, Imai S, Tomita M. Twin pregnancy complicated with bowel strangulation. BMJ Case Rep. 2014 Sep 8;2014:bcr2014205727. doi: 10.1136/bcr-2014-205727. PMID: 25199197; PMCID: PMC4158226.
89. Porter H, Seeho S. Obstructed ileostomy in the third trimester of pregnancy due to compression from the gravid uterus: diagnosis and management. BMJ Case Rep. 2014 Aug 19;2014:bcr2014205884. doi: 10.1136/bcr-2014-205884. PMID: 25139926; PMCID: PMC4139548.
90. Cohen R, Shlomo M, Dil DN, Dinavitser N, Berkovitch M, Koren G. Intestinal obstruction in pregnancy by ondansetron. Reprod Toxicol. 2014 Dec;50:152-3. doi: 10.1016/j.reprotox.2014.10.014. Epub 2014 Oct 24. PMID: 25461913.
91. Gruetter F, Kraljević M, Nebiker CA, Delko T. Internal hernia in late pregnancy after laparoscopic Roux-en-Y gastric bypass. BMJ Case Rep. 2014 Dec 23;2014:bcr2014206770. doi: 10.1136/bcr-2014-206770. PMID: 25538214; PMCID: PMC4275692.
92. Webster PJ, Bailey MA, Wilson J, Burke DA. Small bowel obstruction in pregnancy is a complex surgical problem with a high risk of fetal loss. Ann R Coll Surg Engl. 2015 Jul;97(5):339-44. doi: 10.1308/003588415X14181254789844. PMID: 26264083; PMCID: PMC5096576.
93. Kosai NR, Amin-Tai H, Gendeh HS, Salleh S, Reynu R, Taher MM, Sutton PA, Das S. Pregnant and severe acute abdominal pain: A surgical diagnostic dilemma. Clin Ter. 2015;166(3):110-3. doi: 10.7417/CT.2015.1839. PMID: 26152617.
94. Gudbrand C, Andreasen LA, Boilesen AE. Internal Hernia in Pregnant Women After Gastric Bypass: a Retrospective Register-Based Cohort Study. Obes Surg. 2015 Dec;25(12):2257-62. doi: 10.1007/s11695-015-1693-8. PMID: 26041066.
95. Furderer T, Mantion G, Heyd B. Emergency surgery for intestinal obstruction revealing ileal Crohn's disease (CD) during pregnancy. BMJ Case Rep. 2015 May 12;2015:bcr2013009468. doi: 10.1136/bcr-2013-009468. PMID: 25969480; PMCID: PMC4434334.
96. Vannevel V, Jans G, Bialecka M, Lannoo M, Devlieger R, Van Mieghem T. Internal Herniation in Pregnancy After Gastric Bypass: A Systematic Review. Obstet Gynecol. 2016 Jun;127(6):1013-1020. doi: 10.1097/AOG.0000000000001429. PMID: 27159745.
97. Udigwe GO, Eleje GU, Ihekwoaba EC, Udegbunam OI, Egeonu RO, Okwuosa AO. Acute Intestinal Obstruction Complicating Abdominal Pregnancy: Conservative Management and Successful Outcome. Case Rep Obstet Gynecol. 2016;2016:2576280. doi: 10.1155/2016/2576280. Epub 2016 May 26. PMID: 27313923; PMCID: PMC4899591.
98. Daimon A, Terai Y, Nagayasu Y, Okamoto A, Sano T, Suzuki Y, Kanki K, Fujita D, Ohmichi M. A Case of Intestinal Obstruction in Pregnancy Diagnosed by MRI and Treated by Intravenous Hyperalimentation. Case Rep Obstet Gynecol. 2016;2016:8704035. doi: 10.1155/2016/8704035. Epub 2016 Nov 24. PMID: 27999695; PMCID: PMC5143715.
99. Achour R, Harabi S, Neji K. Spontaneous acute intussusception in a pregnant woman. Case Rep Womens Health. 2016 Dec 12;13:6-8. doi: 10.1016/j.crwh.2016.12.001. PMID: 29593986; PMCID: PMC5863036.
100. Guilbaud T, Bouayed A, Ouaissi M. Emergency reversal of gastric bypass for missed diagnosis of internal hernia and bowel ischemia in a pregnant woman. Surg Obes Relat Dis. 2016 Sep-Oct;12(8):e68-e71. doi: 10.1016/j.soard.2016.08.022. Epub 2016 Aug 18. PMID: 27687910.
101. Joyeux E, Gobenceaux AS, Hoyek T, Dellinger P, Sagot P. Intestinal malrotation complicated by an occlusive syndrome involving internal hernia in a pregnant woman. J Surg Case Rep. 2016 Jul 5;2016(7):rjw113. doi: 10.1093/jscr/rjw113. PMID: 27381017; PMCID: PMC5000836.
102. Mortelmans D, Mannaerts D, Van den Broeck S, Jacquemyn Y, Hubens G. Intussusception in pregnancy after gastric bypass: a case report. Acta Chir Belg. 2016 Dec;116(6):379-382. doi: 10.1080/00015458.2016.1181311. Epub 2016 Jun 2. PMID: 27426666.
103. Narayan B, McCarthy F, Nelson-Piercy C. Chronic anorexia and weight loss due to extensive fibroid compression of the bowel: an unusual complication of uterine fibroids in a patient with a twin pregnancy. BMJ Case Rep. 2016 May 5;2016:bcr2016214858. doi: 10.1136/bcr-2016-214858. PMID: 27151054; PMCID: PMC4885249.
104. Sherer DM, Dalloul M, Schwartzman A, Strasburger A, Farrell RA, Zinn H, Abulafia O. Point-of-care sonographic diagnosis of maternal small bowel obstruction during pregnancy. Ultrasound Obstet Gynecol. 2016 Sep;48(3):403-4. doi: 10.1002/uog.15860. Epub 2016 Aug 9. PMID: 26775812.
105. Yin Y, Li C, Xu C, Wu L, Deng N, Hou H, Wu B. Intestinal obstruction due to congenital malrotation complicating a multiple pregnancy: A rare case report. J Pak Med Assoc. 2017 Feb;67(2):308-310. PMID: 28138191.
106. Boccalatte LA, Achaval Rodríguez J, Beskow A, Cavadas D, Fernando W. Intussusception as a complication of bariatric surgery in pregnant patients: report of one case and revision of the literature. J Surg Case Rep. 2017 Oct 7;2017(10):rjx189. doi: 10.1093/jscr/rjx189. PMID: 29026515; PMCID: PMC5632341.
107. Esterson YB, Villani R, Dela Cruz RA, Friedman B, Grimaldi GM. Small bowel volvulus in pregnancy with associated superior mesenteric artery occlusion. Clin Imaging. 2017 Mar-Apr;42:228-231. doi: 10.1016/j.clinimag.2017.01.002. Epub 2017 Jan 16. PMID: 28126700.
108. Covali R, Ambrosie L, Onofriescu M, Luca A, Dumachita-Sargu G, Aursulesei V, Gafitanu D. Small-Bowel Intussusception in a Pregnant Woman: A Case Report. Perm J. 2017;21:16-179. doi: 10.7812/TPP/16-179. PMID: 28633726; PMCID: PMC5478592.
109. Arapis K, Tammaro P, Goujon G, Becheur H, Augustin P, Marmuse JP. Elevated plasma pancreatic enzyme concentrations after Roux-en-Y gastric bypass may indicate closed loop obstruction. Ann R Coll Surg Engl. 2017 Feb;99(2):e62-e64. doi: 10.1308/rcsann.2016.0334. Epub 2016 Oct 28. PMID: 27791421; PMCID: PMC5392841.
110. Gião Antunes AS, Peixe B, Guerreiro H. Midgut Volvulus as a Complication of Intestinal Malrotation in Pregnancy. ACG Case Rep J. 2017 Jan 18;4:e9. doi: 10.14309/crj.2017.9. PMID: 28144614; PMCID: PMC5247625.
111. Bhadra R, Somasundaram M, Nowak MM, Ravakhah K. A near-fatal case of intussusception and ischaemic perforation of stomach in first-trimester pregnancy: eight years after laparoscopic Roux-en-Y gastric bypass. BMJ Case Rep. 2018 Nov 28;11(1):e226094. doi: 10.1136/bcr-2018-226094. PMID: 30567094; PMCID: PMC6301637.
112. Khan K, Saeed S, Persaud A, Sbeih M, Gray S, Ahmed L. Retrograde jejunojejunal intussusception in a pregnant female after laparoscopic Roux-en-Y gastric bypass. J Surg Case Rep. 2018 May 14;2018(5):rjy094. doi: 10.1093/jscr/rjy094. PMID: 29770188; PMCID: PMC5950921.
113. Kannan U, Gupta R, Gilchrist BF, Kella VN. Laparoscopic management of an internal hernia in a pregnant woman with Roux-en-Y gastric bypass. BMJ Case Rep. 2018 Apr 19;2018:bcr2017221979. doi: 10.1136/bcr-2017-221979. PMID: 29674396; PMCID: PMC5911130.
114. Warsza B, Richter B. Internal Hernia in Pregnant Woman after Roux-en-Y Gastric Bypass Surgery. J Radiol Case Rep. 2018 Jan 31;12(1):9-16. doi: 10.3941/jrcr.v12i1.3257. PMID: 29875982; PMCID: PMC5965396.
115. Bengur FB, Bas M, Aktas A, Yozgatli TK, Bilgin IA, Ozben V, Aytac E, Baca B, Hamzaoglu I, Karahasanoglu T. Laparoscopic management of bowel obstruction due to multiple congenital adhesion bands in pregnancy - a video vignette. Colorectal Dis. 2018 Nov;20(11):1051-1052. doi: 10.1111/codi.14407. Epub 2018 Sep 26. PMID: 30194901.
116. Abu-Zidan FM, Abdel-Kader S, Abusharia MI, Mousa H. Role of magnetic resonance imaging in the management of intestinal obstruction during the first trimester of pregnancy. ANZ J Surg. 2018 Sep;88(9):E683-E684. doi: 10.1111/ans.13655. Epub 2016 Jun 1. PMID: 27246962.
117. Li Z, Song M, Jiang H, Zhou Y. Peutz-Jeghers syndrome complicated with intussusception in late pregnancy. Lancet Oncol. 2019 Dec;20(12):e729. doi: 10.1016/S1470-2045(19)30692-8. PMID: 31797798.
118. Moliere S, Cavillon V, Mesli Y. The case for magnetic resonance imaging in bowel obstruction during pregnancy: Intussusception in a pregnant woman with Roux-en-Y gastric bypass. Am J Obstet Gynecol. 2019 Mar;220(3):282-283. doi: 10.1016/j.ajog.2018.08.010. Epub 2018 Aug 14. PMID: 30118691.
119. Nagata H, Nishizawa H, Mashima S, Shimahara Y. Axial torsion of Meckel's diverticulum causing acute peritonitis in the first trimester of pregnancy: a case report. Surg Case Rep. 2019 Dec 5;5(1):190. doi: 10.1186/s40792-019-0754-y. PMID: 31808013; PMCID: PMC6895344.
120. Hort A, Yoon P, Edye M. Jejuno-anastomotic retrograde intussusception complicating pregnancy after gastric bypass. Surg Obes Relat Dis. 2019 Oct;15(10):1875-1877. doi: 10.1016/j.soard.2019.08.019. Epub 2019 Aug 30. PMID: 31575464.
121. Silva AC, Moreira PS, Simões VC, Sampaio M, Santos MD. Intussusception in a pregnant woman. J Surg Case Rep. 2020 Dec 31;2020(12):rjaa554. doi: 10.1093/jscr/rjaa554. PMID: 33425324; PMCID: PMC7778518.
122. Thomopoulos T, Mantziari S, St-Amour P, Uldry E, Suter M. Management of a Complicated Internal Herniation After Roux-en-Y Gastric Bypass in a 28-Week Pregnant Woman. Obes Surg. 2020 Dec;30(12):5177-5178. doi: 10.1007/s11695-020-04997-0. Epub 2020 Sep 29. PMID: 32996100.
123. Chong E, Liu DS, Rajagopal V, Strugnell N. Midgut volvulus secondary to congenital malrotation in pregnancy. BMJ Case Rep. 2020 May 14;13(5):e234664. doi: 10.1136/bcr-2020-234664. PMID: 32414778; PMCID: PMC7232382.
124. Hsu PK, Yang PY, Su WW, Chen YY. Small bowel obstruction caused by infant compression during pregnancy. Dig Liver Dis. 2020 Nov;52(11):1370-1371. doi: 10.1016/j.dld.2020.03.029. Epub 2020 Apr 17. PMID: 32312672.
125. Kitai T, Yamabe E, Isobe A, Masuhara K, Fukunaga M, Nobunaga T. Successful Laparoscopic Treatment of Small-bowel Obstruction in Early Pregnancy. Gynecol Minim Invasive Ther. 2020 Jul 10;9(4):248-250. doi: 10.4103/GMIT.GMIT_99_19. PMID: 33312873; PMCID: PMC7713660.
126. Bonouvrie DS, Boerma EJ, van Dielen FMH, Leclercq WKG. Internal herniation during pregnancy after banded Roux-en-Y gastric bypass: a unique location. BMJ Case Rep. 2020 Dec 9;13(12):e236798. doi: 10.1136/bcr-2020-236798. PMID: 33298482; PMCID: PMC7733084.
127. Gold D, Nawass M, Imam R, Pillar N, Appelbaum L, Pikarsky A, Khalaileh A, Imam A. Intussusception in a pregnant patient caused by an ectopic pancreatic mass. Clin J Gastroenterol. 2020 Apr;13(2):209-213. doi: 10.1007/s12328-019-01030-5. Epub 2019 Jul 30. PMID: 31364039.
128. Combes AD, Limmer AM, Verschuer K. Small bowel intussusception secondary to Meckel's diverticulum containing polypoid lesion in pregnancy. ANZ J Surg. 2020 Sep;90(9):1774-1776. doi: 10.1111/ans.15626. Epub 2019 Dec 12. PMID: 31833182.
129. Zhao XY, Wang X, Li CQ, Zhang Q, He AQ, Liu G. Intestinal obstruction in pregnancy with reverse rotation of the midgut: A case report. World J Clin Cases. 2020 Aug 26;8(16):3553-3559. doi: 10.12998/wjcc.v8.i16.3553. PMID: 32913863; PMCID: PMC7457094.
130. Yuansheng X, Yi W, Jinyan F. Internal Hernia in Pregnant Woman due to Congenital Transmesenteric Defect. Pak J Med Sci. 2021 Sep-Oct;37(5):1540-1544. doi: 10.12669/pjms.37.5.4116. PMID: 34475945; PMCID: PMC8377930.
131. Latif J, Krivan S, Awan A. Laparoscopic Management of Jejuno-jejunal Intussusception Post Laparoscopic Roux-en-Y Gastric Bypass in a Pregnant Patient. Obes Surg. 2021 Dec;31(12):5504-5505. doi: 10.1007/s11695-021-05689-z. Epub 2021 Sep 1. PMID: 34471995.
132. Chuang MT, Chen TS. Bowel obstruction and perforation during pregnancy: Case report and literature review. Taiwan J Obstet Gynecol. 2021 Sep;60(5):927-930. doi: 10.1016/j.tjog.2021.07.027. PMID: 34507677.
133. Stephens AJ, Wagner SM, Pineles BL, Soto EE. Successful Vaginal Delivery during Acute Small Bowel Obstruction: A Case Report and Review of the Literature. Case Rep Obstet Gynecol. 2021 Mar 3;2021:6632495. doi: 10.1155/2021/6632495. PMID: 33747586; PMCID: PMC7952184.
134. Suminaga Y, Taki M, Okamoto H, Kawamura Y, Sagae Y, Sunada M, Chigusa Y, Horie A, Mandai M, Mogami H. A Case of a Patient with Adhesive Small Bowel Obstruction in Pregnancy after Extensive Myomectomy for Diffuse Uterine Leiomyomatosis. Case Rep Obstet Gynecol. 2022 Sep 26;2022:3601945. doi: 10.1155/2022/3601945. PMID: 36199388; PMCID: PMC9529410.
135. Pavlidi A, Chapusette R, Arvanitakis M. An Unusual Cause of Vomiting in Pregnancy. Gastroenterology. 2023 Oct;165(4):e1-e3. doi: 10.1053/j.gastro.2022.12.025. Epub 2022 Dec 31. PMID: 36592729.
136. Das SS, Ghulam ZA, Al Khitab FH, Juma FIB, Bandok WZM. Redo Gastric Bypass following internal herniation with gangrenous roux limb, in second trimester pregnancy: How safe? Int J Surg Case Rep. 2022 Nov;100:107728. doi: 10.1016/j.ijscr.2022.107728. Epub 2022 Oct 11. PMID: 36270206; PMCID: PMC9586983.
137. Zhao H, Wu L, Yang B, Shang H. Midgut malrotation presenting with hyperemesis gravidarum: A case report. Medicine (Baltimore). 2022 Jul 29;101(30):e29670. doi: 10.1097/MD.0000000000029670. PMID: 35905251; PMCID: PMC9333550.
138. Loukopoulos T, Zikopoulos A, Galani A, Skentou C, Kolibianakis E. Acute intestinal obstruction in pregnancy after previous gastric bypass: A case report. Case Rep Womens Health. 2022 Dec 9;36:e00473. doi: 10.1016/j.crwh.2022.e00473. PMID: 36545008; PMCID: PMC9761365.
139. Lee S, Kim HM, Kang J, Seong WJ, Kim MJ. Fetal intracranial hemorrhage and maternal vitamin K deficiency induced by total parenteral nutrition: A case report. Medicine (Baltimore). 2022 Jan 7;101(1):e28434. doi: 10.1097/MD.0000000000028434. PMID: 35029889; PMCID: PMC8735806.
140. Bhartiya A, Maqsood H, Naeem H, Nazar MW. Reversed intestinal malrotation presenting as bowel obstruction in pregnancy: Case report and literature review. Ann Med Surg (Lond). 2022 Sep 8;82:104570. doi: 10.1016/j.amsu.2022.104570. PMID: 36268437; PMCID: PMC9577447.
141. Scalzo N, Lin ZX, Yick F, Tewari V. Midgut Volvulus in a Pregnant Patient Presenting With Abdominal Pain. ACG Case Rep J. 2023 Feb 9;10(2):e00983. doi: 10.14309/crj.0000000000000983. PMID: 36777462; PMCID: PMC9911195.
142. Wang J, Li Y, Tang T. Intussusception in Late Pregnancy Due to Heterotopic Pancreas. J Gastrointest Surg. 2023 May;27(5):1032-1033. doi: 10.1007/s11605-023-05598-9. Epub 2023 Jan 30. PMID: 36717469.

**Embase and Medline**

1. RINGROSE CA. CHEMICAL SALPINGITIS AND BOWEL OBSTRUCTION FOLLOWING SOAP INJECTION INTO THE GRAVID UTERUS: REPORT OF A CASE WITH CONTINUATION OF THE PREGNANCY AND DELIVERY OF A NORMAL CHILD. Obstet Gynecol. . 196526Cited in: Ovid MEDLINE(R) at http://ovidsp.ovid.com/ovidweb.cgi?T=JS&PAGE=reference&D=med1&NEWS=N&AN=14315336. Accessed December 05, 2023.
2. Banks A.W. A case of Peutz Jeghers’ syndrome in early pregnancy mimicking hyperemesis gravidarum. Aus and NZ J of Obs and Gyn 1974;14:3 (184-186)
3. Holdt D.G, Jacobs A.J, Heidrick W. Intestinal perforation during pregnancy. International J of Gyn and Obs 1982;20:4 (335-339)
4. Docherty P.W, Shedid N.M, Budden G.C. Intestinal obstruction in pregnancy. J of Obs and Gyn. 1983;3:4(216-218)
5. Rachagan S.P, Raman S, Sivanesaratnam V, Sinnathuray T.A. Intestinal obstruction following previous myomectomy and the use of beta-sympathomimetics in pregnancy. European J of Obs Gyn and Repro Biology. 1986;22:1-2 (99-101).
6. Ido S, Lior L, Peter K, Hanan I.S. Small bowel necrosis during pregnancy due to idiopathic internal hernia. J of Pelvic Med and Sur. 2008;14:6 (433-435).
7. Solt I, Kniaz D, Eitan A, Ophir E, Bornstein J. Iatrogenic internal hernia as a cause of small bowel ischemia during pregnancy. J of Gyn Sur 2011;27:2 (119-121).
8. Adamczyk-Gruszka O.K, Lewandowska-Andruszuk I.J, Gluszek S, Gruszka J.A. Mechanical small bowel obstruction as a complication of pregnancy. Med Studies 2015;31:1 (48-51).
9. Mete Ural U, Dagistanli F. A rare cause of acute abdomen in pregnancy: internal herniation. Duzce Medical Journal 2020;22:1 (63-66).
10. Paasch C, Isbruch A, Strik MW, Siegel R. Malignant intestinal obstruction during twin pregnancy: surgical resection of a myxoid liposarcoma without induction of labour. BMJ Case Rep. . 2019;12(7). doi:10.1136/bcr-2019-229742, 10.1136/bcr-2019-229742.
11. Araji T, Wang S, Kandalaft N, Estroff J, Ahmadzia H.K. Recurrent closed loop bowel obstruction in third trimester of pregnancy: case report and review of literature. SN Comprehensive Clin Med. 2022;4:1 Article Number: 178.
